# Curdlan-Based Hydrogels for Potential Application as Dressings for Promotion of Skin Wound Healing—Preliminary In Vitro Studies

**DOI:** 10.3390/ma14092344

**Published:** 2021-04-30

**Authors:** Aleksandra Nurzynska, Katarzyna Klimek, Krzysztof Palka, Łukasz Szajnecki, Grazyna Ginalska

**Affiliations:** 1 Department of Biochemistry and Biotechnology, Medical University of Lublin, Chodzki 1 Street, 20-093 Lublin, Poland; aleksandra.nurzynska@umlub.pl (A.N.); g.ginalska@umlub.pl (G.G.); 2 Faculty of Mechanical Engineering, Lublin University of Technology, Nadbystrzycka 26 Street, 20-618 Lublin, Poland; k.palka@pollub.pl; 3 Department of Polymer Chemistry, Maria Curie-Skłodowska University in Lublin, M. Curie-Skłodowska Sq. 2, 20-031 Lublin, Poland; l.szajnecki@umcs.pl

**Keywords:** biocompatibility, calcium ions, chronic wounds, hydrogels, skin fibroblasts, wound exudate, bioactive wound dressing

## Abstract

The aim of this work was to establish whether novel curdlan-based hydrogels enriched with Ca^2+^ ions may be considered as potential candidates for dressings, for the acceleration of skin wound healing. Firstly, biomaterials were allocated for evaluation of structural and mechanical properties. Subsequently, the ability of hydrogels to absorb simulated wound fluid and water vapor permeability, as well their capacity to release calcium ions, was evaluated. The biocompatibility of biomaterials was assessed using normal human skin fibroblasts. Importantly, the main features of the obtained curdlan-based hydrogels were compared with those of KALTOSTAT^®^ (a commercial calcium sodium alginate wound dressing). The obtained results showed that curdlan-based biomaterials possessed a mesoporous structure (pore diameter ranged from 14–48 nm) and exhibited a good ability to absorb simulated wound fluid (swelling ratio close to 974–1229%). Moreover, in a wet state, they enabled proper water vapor transmission rate (>2000 g/m^2^/day), thanks to their hydrogel structure. Finally, it was found that biomaterial composed of 11 wt.% of curdlan (Cur_11%) possessed the most desirable biological properties in vitro. It released a beneficial amount of calcium ions to the aqueous environment (approximately 6.12 mM), which significantly enhanced fibroblast viability and proliferation. Taking into account the beneficial properties of Cur_11% biomaterial, it seems justified to subject it to more advanced cell culture experiments in vitro and to in vivo studies in order to determine its precise influence on skin wound healing.

## 1. Introduction

Hard-to-heal wounds (mainly the chronic ones) still constitute a huge problem for current medicine [1,2,3]. It has been indicated that over 20 million people worldwide suffer from this ailment. Such wounds are associated with many inconveniences for patients, such as reduction of their quality of life, physical limitations, numerous hospital stays, and the need for frequent dressing changes. It is estimated that the global wound care market will consume $22 billion dollars in 2024 [4]. Treatment of hard-to-heal wounds with bioactive dressings is crucial to decrease the cost of therapy, increase the quality of patient life, and accelerate wound healing [5,6,7,8].

Appropriate dressings for promoting skin wound healing must meet many requirements. They should primarily have the ability to stop bleeding and to absorb a high amount of wound exudate. A moist environment (but not a wet one) at the wound site is considered to be a pivotal condition for the proper healing process [3,5,6,9,10]. Dressings maintaining moisture decrease the risk of scar formation, enhance re-epithelialization, and facilitate cell migration into the wound. They also support cell viability and proliferation [5,6,7]. Furthermore, the appropriate dressings cannot strongly adhere to the wound site in order to enable the formation of new tissue without trauma that often occurs during repeated dressing changes [3,5,6,7,9,10]. It is well known that wound dressings constitute a barrier to external contamination. Therefore, they should exhibit mechanical stability during application, which allows for their wearing in a suitable manner, and then easy removal. At the same time, wound dressings should exhibit elastic texture to adapt to the wound bed and should be flexible while the patient is moving [5,11,12,13,14]. Permeability of gases is another important feature of wound dressings. Such biomaterials should allow skin to “breathe”. Hence, they must enable the exchange of gases, such as CO_2_, O_2_, and H_2_O [3,5,6,7]. On the other hand, biocompatibility is a mandatory property of biomaterials [15,16]. Modern dressings should not only be non-toxic, but they should also accelerate the healing process [3,6]. Because various types of cells (*inter alia* monocytes, fibroblasts, keratinocytes) are involved in the healing process, desirable wound dressings should enhance their viability and proliferation, as well as migration [5,6]. In turn, considering the economic aspects, suitable dressings should not be too expensive in order to avoid the high cost of treatment [17,18,19]. Taking into account all the aforementioned criteria, hydrogels, especially those made from natural polymers, have attracted notable attention in the field of tissue engineering [3,11,20,21,22,23,24,25]. They are fabricated using various techniques, such as phase separation, solvent evaporation, porogen leaching, electrospinning, freeze-drying, or supercritical CO_2_ drying, which allows for obtaining of porous biomaterials with a high ability to absorb wound exudate [6,26,27].

Curdlan is an unbranched, bacterial β-1,3-glucan, which possesses beneficial chemical, rheological, and biological properties [28,29]. It is colorless, tasteless, and odorless. Curdlan is not soluble in water (it forms aqueous suspension), but this polysaccharide is soluble in alkaline solutions [16,30]. It also possesses the ability to form non-toxic, flexible but solid hydrogels, when both its aqueous suspension is heated to 55–90 °C and when its alkaline solution is subjected to ion-exchanging dialysis. Thanks to all the aforementioned features, it is successfully used for tissue engineering applications [15,16,29]. It is also worth highlighting that curdlan belongs to the family of β-glucans, which exhibit immunostimulatory effects [16,28,30]. Therefore, β-glucans are considered as suitable wound healing agents. They are known to promote wound healing by increasing the infiltration of macrophages, which results in better tissue granulation and collagen deposition, as well as re-epithelialization. Moreover, β-glucans are characterized by good stability and resistance to wound proteases [31]. Nevertheless, the potential of curdlan as a component of wound dressings is poorly investigated. Basha et al. [32] demonstrated that curdlan/polyvinyl alcohol (PVA) nanofibrous scaffolds possess good swelling behavior and are non-toxic towards myoblast cells (L6 cells). Wojcik et al. [33] fabricated biocompatible curdlan/agarose and curdlan/chitosan dressings with superabsorbent ability. In turn, our recent study demonstrated that curdlan-based biomaterials enriched with copper ions possess antibacterial activity but simultaneously exhibit cytotoxicity towards fibroblast cells [34]. The aim of the current work was to determine whether curdlan-based hydrogels enriched with calcium ions are suitable candidates as dressings for the acceleration of wound healing. The biomaterials were prepared using a new procedure, which combined ion-exchanging dialysis against calcium chloride (CaCl_2_) solution, followed by two-step freezing with subsequent freeze-drying. It is worth underlining that the fabrication procedure of the presented curdlan biomaterials was described in the Polish patent application no. 432848, “the fabrication method of absorbent biomaterial based on curdlan for medical applications”. The obtained biomaterials were subjected to evaluation of their structural, mechanical, and in vitro biological properties. Importantly, the main properties of novel curdlan-based hydrogels were compared with those of KALTOSTAT^®^ (commercial calcium sodium alginate wound dressing).

## 2. Materials and Methods

### 2.1. Materials

Bovine serum albumin (BSA), formaldehyde solution (36.5–38%), Hoechst 33342 fluorescent dye, a Live/Dead Cell Double Staining Kit, penicillin-streptomycin solution, phosphate buffered saline (PBS), silica gel with indicator (orange gel), sodium dodecyl sulfate (SDS), thiazolyl blue tetrazolium bromide (MTT), tris-hydroxymethyl aminomethane (TRIS), triton X-100, and trypsin-EDTA solution (0.25%) were obtained from Sigma-Aldrich Chemicals, Warsaw, Poland. Curdlan (MW 80 kDa) was purchased from Wako pure Chemicals Industries, Osaka, Japan, whereas the fetal bovine serum (FBS) was from Pan-Biotech, Aidenbach, Germany. Eagle’s Minimum Essential Medium (EMEM) and normal human skin fibroblasts (BJ cell line, CRL-2522^TM^) were supplied by ATTC, Teddington, UK, while AlexaFluor^TM^ 635 Phalloidin was supplied by Invitrogen, Warsaw, Poland. Calcium chloride (CaCl_2_), dimethyl sulfoxide (DMSO), sodium chloride (NaCl), hydrochloric acid (HCl), and sodium hydroxide (NaOH) were purchased from Avantor Performance Materials, Gliwice, Poland. A calcium ion detection kit (Calcium CPC) was supplied by BioMaxima, Lublin, Poland. Calcium sodium alginate dressing—KALTOSTAT^®^ (10 cm × 20 cm) was obtained from ConvaTec, Tredegar, UK. Before experiments, a KALTOSTAT^®^ sheet was cut into small discs (2.2 cm in diameter) and sterilized using ethylene oxide.

### 2.2. Preparation of Curdlan-Based Biomaterials

**Figure 1:**
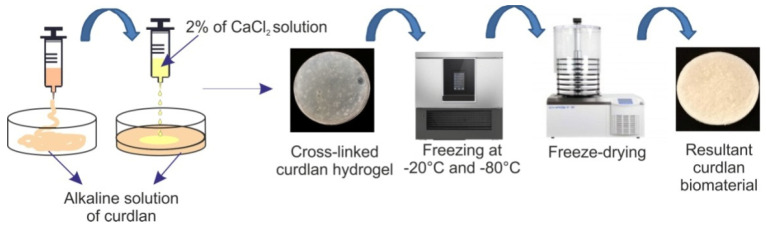
Schematic illustration presenting the general fabrication procedure of curdlan-based biomaterials, according to description in Polish patent application no. 432828.

The curdlan-based biomaterials were fabricated according to the procedure described in the Polish patent application no. 432848 (“the fabrication method of absorbent biomaterial based on curdlan for medical applications”). In the first step, solutions containing 5 wt.%, 8 wt.%, and 11 wt.% of curdlan in 0.3 M aqueous NaOH were prepared using a magnetic stirrer (25 °C, 40 rpm, Cimarec+^TM^ Stirrer, ThermoFisher Scientific, Waltham, MA, USA). These solutions were placed into round-shaped forms (2.2 cm in diameter) and then were allocated to ion-exchanging dialysis against 2% of CaCl_2_ solution (3 h, 25 °C). Curdlan cross-linking by Ca^2+^ ions was undergone in accordance with the mechanism described in detail by Sato et al. [35]. Briefly, during the dialysis process, Na^+^ and OH^−^ ions outflow outside from of the curdlan solution, which leads to a change of the pH and, as a consequence, to a conformational transition of curdlan molecules—from random coil to triple helix. Simultaneously, Ca^2+^ ions inflow to the curdlan solution and cross-link the helical molecules of this polysaccharide (the curdlan-based biomaterial). After dialysis, the obtained solid hydrogels were rinsed with deionized water (three times for 15 min in order to remove residues of calcium chloride) and placed in a freezer at −20 °C for two days, followed by −80 °C for 2 h. Finally, they were freeze-dried (LYO GT2-Basic, SRK Systemtechnik GmbH, Riedstadt, Germany) for 24 h. The general fabrication procedure is presented in Figure 1. Resultant dry biomaterials were denoted: Cur_5%, Cur_8%, and Cur_11%. Before experiments, these biomaterials were sterilized using ethylene oxide.

### 2.3. Assessment of Biomaterial Morphology

The morphology of the biomaterial specimens was evaluated using a scanning electron microscope (Nova NanoSEM 450, FEI, Oxford, UK) equipped with an Octane Pro EDS detector (EDAX), which enabled chemical analysis and identification of occurring precipitates.

### 2.4. Evaluation of Specific Surface Area, Pore Distribution, and Pore Diameter of Biomaterials

For this experiment, curdlan-based biomaterials were prepared as cylindrical samples (5 mm in diameter and 10 mm in length). The specific surface area (SSA) of biomaterials was determined by the Brunauer-Emmett-Teller (BET) method from N_2_ adsorption isotherms obtained at −196 °C using ASAP 2020 HD (Micromeritics Inc., Norcross, GA, USA). Before analyses, the biomaterial samples were degassed at 25 °C for 16 h and then under vacuum (10^−5^ to 10^−6^ mmHg) at 25 °C for 5 h. The original density functional theory (DFT) and classical thermodynamic Barrett-Joyner-Halenda (BJH) theory were used for calculation of pore size distribution and average pore diameter, respectively.

### 2.5. Wound Fluid Uptake Test

In order to assess the ability of biomaterials to absorb wound exudate, the experiment was carried out using simulated wound fluid (SWF), according to a procedure described previously [34]. Briefly, curdlan-based biomaterials and KALTOSTAT^®^ samples were weighed in the dry state and then immersed in SWF (room temperature). The SWF solution was prepared immediately before the experiment using 2.22 g of CaCl_2_, 23.38 g of NaCl, 9.69 g of TRIS, and 20 g of BSA per 1 L of deionized water. The final pH of the obtained solution was 7.5. At fixed time intervals, the specimens were taken out of solution, blotted with tissue paper, re-weighted, and re-immersed. The experiment was performed for 24 h. The swelling ratio was determined using the following Equation (1):

(1) $$SW\;\left(\%\right)=\frac{\left(Ws-Wd\right)}{Wd}\times 100$$

where *Ws* denotes the weight of swollen biomaterials (g) and *Wd* denotes the weight of dry ones (g).

### 2.6. Mechanical Properties

The mechanical properties, namely Young’s modulus (E) and tensile strength (TS), were evaluated according to ASTM D638-14 standard recommendations [36]. Curdlan-based biomaterials were prepared as dumbbell-shaped strips (Type V) in accordance with procedure described in Section 2.2. Prior to test, the samples of curdlan-based biomaterials were soaked in SWF for 24 h (room temperature), to mimic conditions after absorption of wound exudate. Then, the biomaterials were measured using an Autograph AG-X Plus (Shimadzu, Kioto, Japan) testing machine (preload value of 1N, crosshead moving speed 10 mm/min followed by basic load rate 0.5 mm/min). Measurements of Young’s modulus were carried out using a non-contact extensometer (TRViewX120S, Shimadzu, Kioto, Japan). In turn, mechanical properties of KALTOSTAT^®^ biomaterial were not determined because of its unstable structure in a wet state.

### 2.7. Water Vapor Transmission Test

The water vapor transmission test was carried out according to a procedure described previously [34]. For this purpose, silica gel with indicator (5 g) was put into glass vials (diameter of mouth was 1 cm) and they were subjected to drying (Drying oven SUP-65, Wamed, Warsaw, Poland). Simultaneously, the samples of curdlan-based biomaterials and KALTOSTAT^®^ specimens were immersed in SWF (24 h, room temperature). Then, the vials containing dry silica gel were weighed. The biomaterials were removed from the solution, blotted with tissue paper, and mounted precisely on the mouth of the vials. After 24 h incubation at 37 °C and 95% relative humidity (Heraeus cytoperm 2, Thermo Scientific, Waltham, MA, USA), the biomaterials were removed from the vials and the mass of vials with wet silica gel was measured. The water vapor transmission rate (WVTR) was calculated as follows:

(2) $$WVTR\;\left(g/m^{2}/day\right)=\frac{Ww-Wd}{S}$$

where *Ww* denotes the weight of the vial with wet gel (g), *Wd* denotes the weight of the vial with dry gel (g), and *S* denotes the surface of the vial mouth (m^2^).

### 2.8. Calcium Ion-Releasing Ability

To evaluate the capacity of biomaterials to release calcium ions in an aqueous environment, the liquid extracts from specimens were prepared according to ISO 10993-5:2009 standard recommendations [37]. The samples of curdlan-based biomaterials and KALTOSTAT^®^ specimens were placed into a 12-well plate and EMEM medium with an addition of 2% FBS was added (extraction ratio was equal to 0.1 g of biomaterial/1 mL of EMEM medium). After 24 h incubation at 37 °C in a humidified atmosphere of 5% CO_2_ and 95% air (Heraeus cytoperm 2, Thermo Scientific, Waltham, MA, USA), the liquid extracts were collected. The EMEM medium incubated without biomaterials served as a control extract. The concentration of calcium ions in collected solutions was evaluated using the Calcium ion detection kit in accordance with the manufacturer’s protocol.

### 2.9. Cell Culture Experiments

The cell culture experiments were performed using normal human skin fibroblasts, i.e., BJ cell line (ATCC, London, UK), as it is known as a good model for the evaluation of wound healing in vitro [33,38,39,40,41]. The cells were grown in EMEM medium with an addition of 10% FBS, 100 U/mL penicillin, and 100 μg/mL streptomycin. According to ATCC directions, BJ cells were cultured at 37 °C in a humidified atmosphere of 5% CO_2_ and 95% air (Heraeus cytoperm 2, Thermo Scientific, Waltham, MA, USA). To assess fibroblast response to the tested biomaterials, the liquid extracts from the samples of curdlan-based biomaterials and KALTOSTAT^®^ specimens were prepared according to ISO 10993-5:2009 standard directions [37], as described in Section 2.8. For evaluation of cell viability, extracts were prepared using EMEM supplemented with 2% FBS, while extracts obtained in EMEM with an addition of 10% FBS was applied for estimation of cell proliferation. As a control extract, appropriate EMEM medium (with 2% or 10% FBS) incubated without biomaterials was utilized. The ISO 10993-5:2009 standard guidelines [37] are commonly applied for the evaluation of biological properties in vitro of biomaterials with biomedical potential [33,34,42,43,44].

#### 2.9.1. Cell Viability

In the first step, BJ cells were seeded in 96-well plates in 100 μL of EMEM medium with an addition of 10% FBS at a high concentration equal to 1.5 × 10^4^ cells/well. After overnight incubation, the culture medium was replaced with appropriate liquid extracts (obtained in EMEM with 2% FBS—to minimize cell proliferation) and the plates were incubated for 24 h. Furthermore, 10% dimethyl sulfoxide (DMSO) solution in culture medium was served as positive control of cytotoxicity. The fibroblast viability was assessed quantitatively via MTT test (as described in details earlier [45]) as well as qualitatively using the Live/Dead Double Staining Kit in accordance with manufacturer protocol. The fibroblasts stained with fluorescent dyes were observed under confocal laser scanning microscope (CLSM, Olympus Fluoview equipped with FV1000, Shinjuku, Japan).

#### 2.9.2. Cell Proliferation

Based on results obtained during cell viability evaluation, Cur_11% biomaterial was selected and its influence on fibroblast proliferation was assessed. The BJ fibroblasts were seeded in 96-well plates in 100 μL of EMEM medium with an addition of 10% FBS at low concentration equal to 2 × 10^3^ cells/well. After overnight incubation, culture medium was replaced with biomaterial liquid extracts (obtained in EMEM with 10% FBS—to allow normal cell proliferation) and the plates were incubated for three and five days. Culture medium incubated without biomaterial was served as a control. After incubation time, the cell proliferation was assessed by the WST-8 test. The fold increase in cell proliferation was calculated using the following Equation (3):

(3) $$Fold\;increase\;in\;cell\;proliferation=\frac{B-A}{A}$$

where *A* denotes the average value of optical density (OD) obtained for the tested group at day three, while *B* denotes the average value of OD obtained for the tested group at day five.

Moreover, the cells were stained with Hoechst 33342 and AlexaFluor^TM^ 635 Phalloidin fluorescent dyes and observed under CLSM (Olympus Fluoview equipped with FV1000, Shinjuku, Japan).

### 2.10. Statistical Analysis

Most of the analyses were performed at least in three independent experiments and obtained results have been shown as mean values ± standard deviation (SD). For determination of statistical differences between samples (*p* < 0.05), the unpaired *t*-test or one-way ANOVA test followed by Tukey’s multiple comparison test were used (GraphPad Prism 5, Version 5.04 Software).

## 3. Results and Discussion

### 3.1. Morphology of Biomaterials

**Figure 2:**
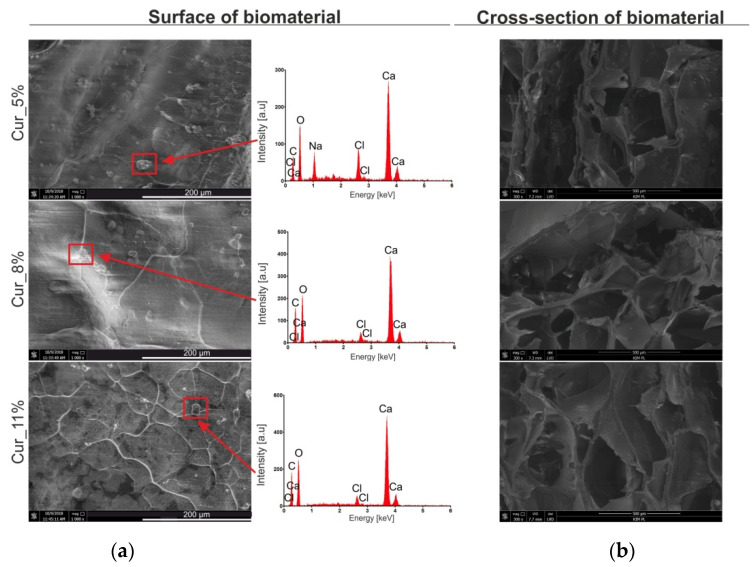
SEM images of Cur_5%, Cur_8%, and Cur_11% biomaterials and EDS spectra of visible precipitates. Magnification of SEM images: (**a**) 1000× (surface of biomaterials) and (**b**) 300× (internal cross-section of biomaterials).

The scanning electron microscope (SEM) images (Figure 2a) showed that surfaces of all tested curdlan-based samples were covered with sporadically visible precipitates (examples of such precipitates have been marked in red frames). They were primarily composed of calcium and chlorine, as proven by energy dispersive spectroscopy (EDS) spectra (Figure 2a). Most likely, calcium chloride precipitates occurring on biomaterial surfaces should have the ability to dissolve during direct contact with wound exudate. As a consequence, this would lead to an increase in calcium ion concentration at the wound site. This is a very desirable phenomenon because, after skin injury, the concentration of Ca^2+^ ions gradually increases, which enhances wound healing [46,47,48]. It was experimentally proved (in vitro and in vivo) that wound dressings possessing the ability to release calcium ions into the wound site promote hemostasis, enhance cell proliferation, and accelerate the formation of granulation tissue [6,20,47,48,49,50,51,52,53].

Moreover, SEM images of cross-sections of the biomaterials revealed that they had a porous internal structure (Figure 2b). It is worth pointing out that the use of porous dressings is beneficial for the wound healing process. On the one hand, porosity positively affects the ability of biomaterials to absorb liquids, because porous samples—thanks to higher specific surface area—possess better swelling capacity compared to non-porous ones [54,55]. On the other hand, occurrence of pores within the biomaterial structure allows for the exchange of gases and nutrients, which are necessary for cells [26,56].

### 3.2. Specific Surface Area, Pore Distribution, and Pore Diameter of Biomaterials

Specific surface area (SSA), pore distribution, and pore diameter are key features of porous biomaterials. The SSA is mostly expressed as a ratio between total surface area of a sample and its weight. The value of SSA strictly depends on the size of pores. The pores can be classified as micropores (diameter < 2 nm), mesopores (diameter 2–50 nm), or macropores (diameter > 50 nm), wherein the presence of micropores mainly determines the value of a specific surface area. It was shown that microporous and mesoporous biomaterials have very good or good absorption ability, due to high specific surface area and high pore volume. In turn, macroporous samples possess relatively poor swelling ability [57,58,59,60,61,62].

**Figure 3:**
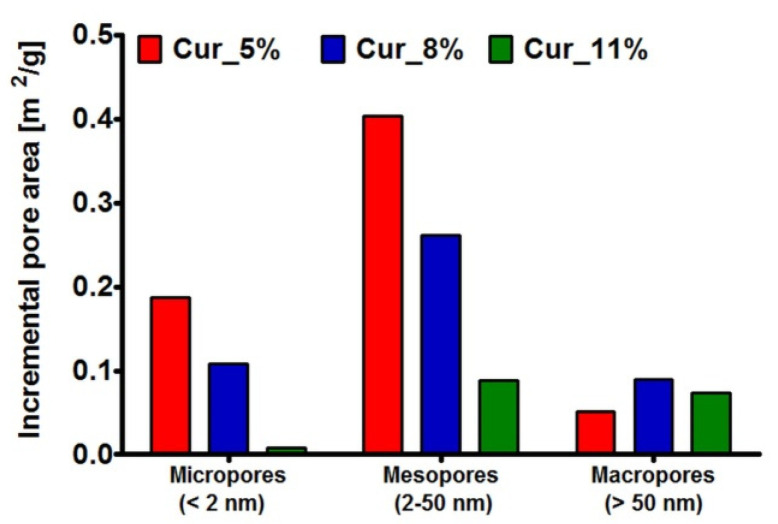
Distribution and pore size within Cur_5%, Cur_8%, and Cur_11% biomaterials.

In the case of curdlan-based biomaterials, it was demonstrated that the specific surface area and cumulative pore area of samples increased in the following order: Cur_5% > Cur_8% > Cur_11% (Table 1). Thus, as expected, Cur_5% and Cur_8% biomaterials were mainly characterized by the presence of micropores and mesopores, while Cur_11% possessed predominantly mesopores and macropores (Figure 3). Interestingly, because the fabrication method of tested biomaterials was the same, it seems that concentration of curdlan in samples affected their architecture. Some researchers demonstrated that biomaterial porosity decreased along with the increase of polymer concentration due to a higher density in samples [63,64,65,66]. Thus, these results can explain our observations. Despite some differences between samples, all tested curdlan-based biomaterials can be classified as mesoporous (based on average pore size values, Table 1). Given the values of specific surface area for curdlan-based biomaterials (50.13–100 × 10^−2^ m^2^/g; Table 1), it seems that they exhibited moderate parameters. For instance, Chen et al. [59] fabricated polyurethane membranes as potential wound dressings. The authors indicated that their specific surface area ranged from 230 to 270 × 10^−2^ m^2^/g. In turn, Zhang et al. [67] prepared three types of porous alginate wound dressings. The specific surface area of these materials ranged from 21.77 to 45.44 × 10^−2^ m^2^/g.

### 3.3. Ability of Biomaterials to Swell in Wound Fluid

**Figure 4:**
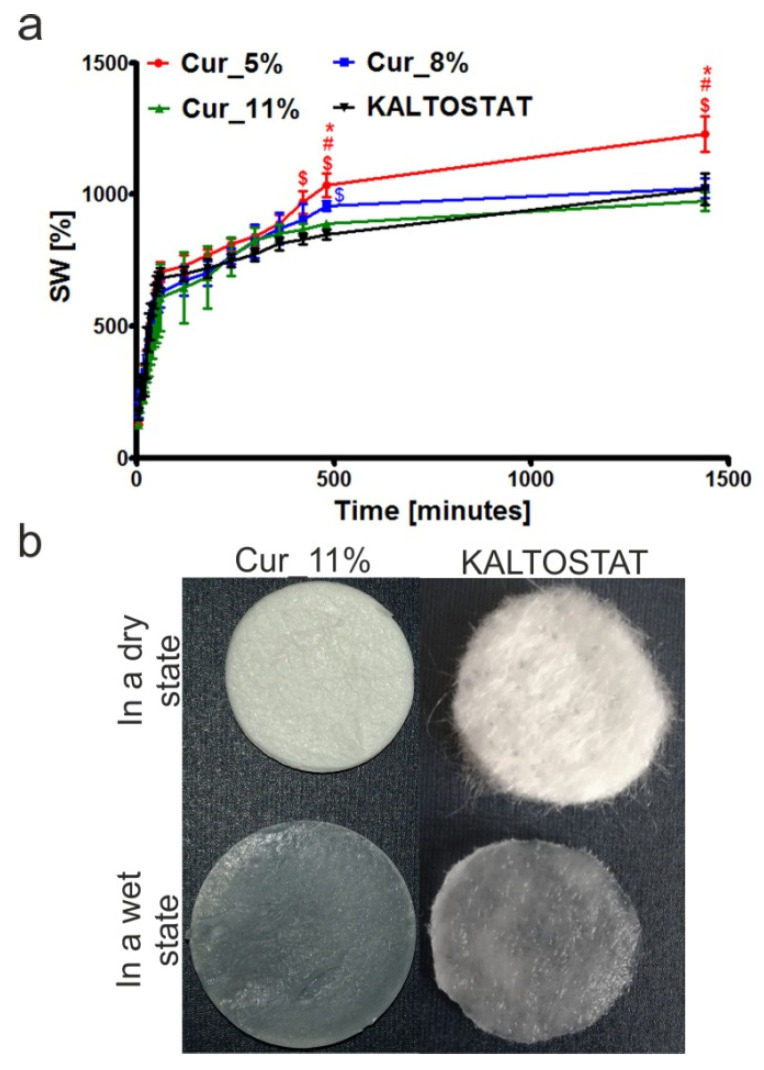
The ability of Cur_5%, Cur_8%, Cur_11%, and KALTOSTAT^®^ biomaterials to absorb simulated wound fluid (SWF) after 24 h incubation (**a**) (**^#^** significantly different results compared to Cur_8% biomaterial; * significantly different results compared to Cur_11% biomaterial; **^$^** significantly different results compared to KALTOSTAT^®^; one-way ANOVA test followed by Tukey’s multiple comparison, *p* < 0.05). Representative images presenting Cur_11% and KALTOSTAT^®^ biomaterials in a dry state—before incubation in SWF and in a wet state—after incubation in SWF (**b**).

The measurement of sample weights before and after their incubation in simulated wound fluid (SWF) showed that all tested biomaterials possessed good ability to absorb liquid (Figure 4a).

After 24 h incubation in SWF, the swelling ratio (SW) of Cur_5%, Cur_8%, Cur_11%, and KALTOSTAT^®^ biomaterials was 1229 ± 66.59%, 1025 ± 36.31%, 974 ± 35.56%, and 1021 ± 59.19%, respectively. These results confirmed the data obtained from BET theory (Table 1), as the swelling ability of curdlan-based biomaterials decreased along with the decline of their specific surface area. Importantly, all tested curdlan-based biomaterials exhibited swelling ability at least as good as KALTOSTAT^®^. Because this commercial dressing is designed for moderately to heavily exuding wounds (based on manufacturer data from [68]), it seems that all fabricated curdlan-based biomaterials may be considered as promising absorbent dressings.

Moreover, it is worth underlining that, in contrast to the loose, fibrous-like morphology of KALTOSTAT^®^ in a dry state, the fabricated curdlan-based biomaterials possessed a compact, foam-like structure. When all tested samples (curdlan-based and KALTOSTAT^®^) were swollen in SWF, they became semi-transparent hydrogel structures. The representative pictures of Cur_11% and KALTOSTAT^®^ biomaterials in dry and wet states have been presented in Figure 4b. Hydrogel biomaterials have many properties of ideal wound dressings. They are semi-transparent or transparent, allowing for wound observation without removal of the dressing and they have the ability to maintain a moist environment, which enhances wound healing [5,6,11]. Furthermore, such dressings are known to reduce pain for patients, because they provide a cooling effect. Hydrogels also exhibit low adherence to the wound site, which decreases pain and overcomes trauma during dressing changes [5,6].

### 3.4. Mechanical Properties of Biomaterials

The swollen curdlan-based hydrogels possessed a compact structure and, simultaneously, they were flexible and could also fit into the site where they were placed (data not shown). The mechanical test was conducted for wet biomaterials and the results have been summarized in Table 2.

It was shown that the higher the curdlan concentration in the sample, the better the mechanical properties. Thus, Cur_11% had the highest values of Young’s modulus (0.296 ± 0.036 MPa) and tensile strength (0.051 ± 0.008 MPa), which indicates its higher stability compared to the other tested biomaterials. This observation is in accordance with results presented by other researchers, as the hardness and stiffness of the biomaterials increased with greater concentration of polymer in the sample [69,70].

### 3.5. Water Vapor Permeability of Biomaterials

**Figure 5:**
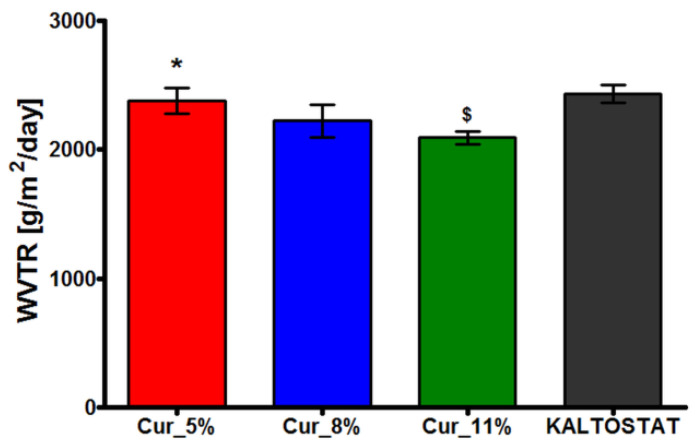
Water vapor transmission rate (WVTR) of Cur_5%, Cur_8%, Cur_11%, and KALTOSTAT^®^ biomaterials. (* significantly different results compared to Cur_11% hydrogel; ^$^ significantly different results compared to KALTOSTAT^®^ biomaterial; one-way ANOVA followed by Tukey’s multiple comparison test, *p* < 0.05).

The performed experiment showed that all curdlan-based hydrogels and KALTOSTAT^®^ biomaterial were permeable to water vapor with water vapor transmission rate (WVTR) values above 2000 g/m^2^/day (Figure 5).

The WVTR values for Cur_5%, Cur_8%, Cur_11%, and KALTOSTAT^®^ biomaterials were 2377 ± 98.23 g/m^2^/day, 2223 ± 124.8 g/m^2^/day, 2091 ± 48.41 g/m^2^/day, and 2434 ± 70.71 g/m^2^/day, respectively. It is considered that evaluation of WVTR is crucial for determination of the biomedical potential of future dressings [14,71]. If WVTR of biomaterials are too low, this can result in exudate accumulation which, as a consequence, may inhibit the healing process and increase risk of infection [72]. The WVTR of normal skin is equal to 204 g/m^2^/day, while injured skin possesses significantly higher permeability with WVTR ranging from 279 g/m^2^/day to 5138 g/m^2^/day [71]. An “ideal” dressing should reduce excessive dehydration of skin as well as preventing exudate buildup. Thus, its WVTR should be close to 2000–2500 g/m^2^/day [34,71,73]. The results obtained for curdlan-based biomaterials and KALTOSTAT^®^ dressing indicate that they should allow for proper water vapor permeability and should maintain a moist environment at the wound site without undue dehydration.

### 3.6. Ability of Biomaterials to Release Calcium Ions

**Figure 6:**
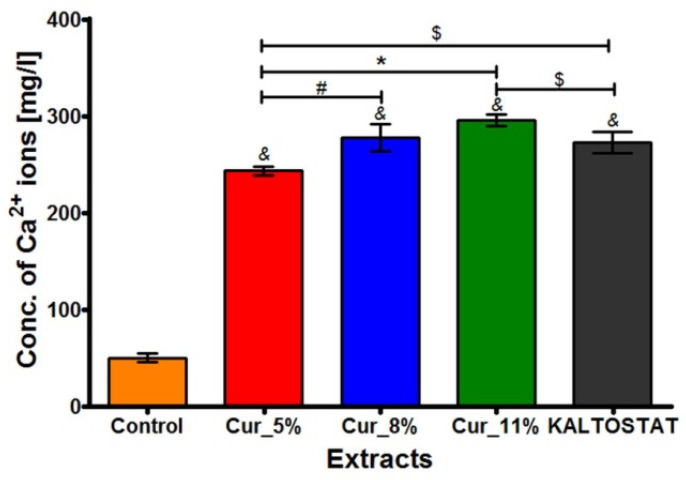
Concentration of calcium ions released from Cur_5%, Cur_8%, Cur_11%, and KALTOSTAT^®^ biomaterials after 24 h incubation in culture medium (EMEM with addition of 2% FBS). Extracts were prepared according to ISO 10993-5:2009 standard recommendations [37]. (^&^ significantly different results compared to control extract—culture medium incubated without biomaterials; ^#^ significantly different results compared to Cur_8% extract; * significantly different results compared to Cur_11% extract; ^$^ significantly different results compared to KALTOSTAT^®^ extract; one-way ANOVA followed by Tukey’s multiple comparison test, *p* < 0.05).

The calcium ion detection kit revealed that all the tested biomaterials possessed a huge capacity to release Ca^2+^ ions to the culture medium (Figure 6). After 24 h incubation, the concentrations of Ca^2+^ ions in extracts obtained from Cur_5%, Cur_8%, Cur_11%, and KALTOSTAT^®^ biomaterials were approximately five- to six-fold higher (*p* < 0.05) than the concentrations of these ions in the control extract (culture medium incubated without biomaterials). Of the tested biomaterials, Cur_11% released the greatest amount of Ca^2+^ ions.

It is worth underlining that calcium ions (Ca^2+^ ions) play a pivotal role in wound healing [46,47,48]. Immediately after skin injury, the concentration of these ions gradually increases, while the highest level is observed during the inflammation and proliferative phases. Generally, it was found that the concentration of calcium ions during wound healing increases from 0.5 mM (basal layer of skin) to above 1.4 mM (in the stratum granulosum). The Ca^2+^ ions are crucial for the formation of fibrin clots as they constitute clotting factor IV. They are released by platelets and their presence stimulates the synthesis and release of other clotting factors (i.e., VII, IX, and X). They also promote conversion of prothrombin to thrombin. During the next two phases (inflammation and proliferation), Ca^2+^ ions induce an influx of inflammatory cells, fibroblasts, and keratinocytes. They also regulate proliferation of fibroblasts and keratinocytes [46,53,74,75,76]. Thus, given the high significance of calcium ions in early phases of wound healing, some bioactive dressings for the treatment of chronic wounds (e.g., KALTOSTAT^®^) have the ability to release Ca^2+^, which significantly accelerates skin regeneration [3,7,20,77]. In our study, we demonstrated that after 24 h incubation, the concentration of calcium ions released from tested samples to the culture medium was approx. 4.82–6.12 mM for curdlan-based biomaterials and approx. 5.72 mM for KALTOSTAT^®^. These results seem to be promising when compared with data obtained by Ågren [50]. The author evaluated the biomedical potential of four commercial alginate dressings (ALGOSTERIL, Comfeel Alginate, KALTOSTAT, and SORBSAN), as well as polyurethane film dressing (control sample) using a wound model in domestic pigs. After 24 h treatment with alginate-based dressings and polyurethane film, the concentration of calcium ions in the collected wound exudate was 5.0–11.7 mM and 2.8 mM, respectively. Moreover, it was shown that all alginate-based biomaterials enhanced re-epithelialization of pig skin significantly better, compared to control (polyurethane film dressing). Thus, these results may suggest that curdlan-based biomaterials release a suitable amount of calcium ions to ensure proper wound healing.

### 3.7. Fibroblast Viability

**Figure 7:**
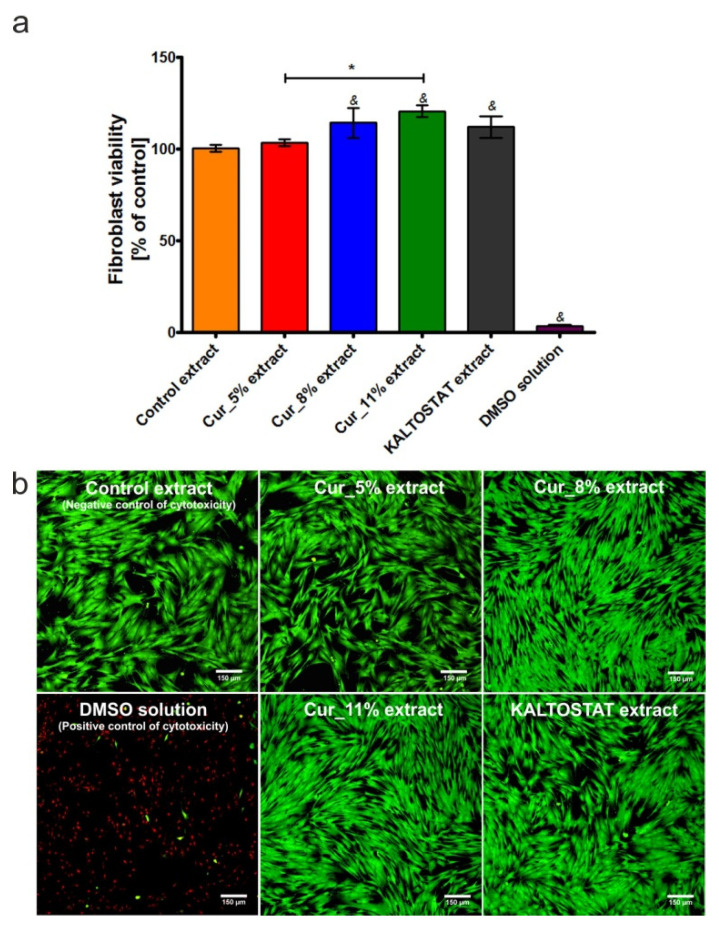
Fibroblast viability (BJ cell line, ATCC CRL-2522^TM^) after 24 h incubation with extracts obtained from Cur_5%, Cur_8%, Cur_11%, and KALTOSTAT^®^ biomaterials. Extracts were prepared in culture medium (EMEM with addition of 2% FBS) according to ISO 10993-5:2009 standard recommendations [37]. Culture medium incubated without biomaterials (control extract) and 10% DMSO solution were used as negative and positive controls of cytotoxicity, respectively. The results obtained using the MTT assay (**a**) (^&^ significantly different results compared to culture medium incubated without biomaterials (control extract); * significantly different results compared to Cur_11% extract; one-way ANOVA followed by Tukey’s multiple comparison test, *p* < 0.05). The confocal laser scanning microscope (CLSM) images presenting viable cells (green fluorescence) and dead cells (red fluorescence) after staining with Live/Dead Cell Double Staining Kit (**b**); magnification 100x, scale bar equals 150 μm.

The thiazolyl blue tetrazolium bromide (MTT) assay demonstrated that extracts obtained from Cur_5%, Cur_8%, Cur_11%, and KALTOSTAT^®^ were not only non-toxic towards BJ fibroblasts, but also supported viability of these cells (Figure 7a). Thus, after 24 h incubation, the viability of BJ cells treated with extracts from Cur_5%, Cur_8%, Cur_11%, and KALTOSTAT^®^ was 103.30 ± 1.91%, 114.30 ± 8.14%, 120.60 ± 3.17%, and 112.70 ± 5.85%, respectively, in comparison with the viability of cells grown in the control extract (culture medium incubated without biomaterials, i.e., negative control of cytotoxicity). The confocal laser scanning microscope (CLSM) observations confirmed the results obtained with the MTT test. The fibroblast cells treated with the control extract and extracts from Cur_5%, Cur_8%, Cur_11%, and KALTOSTAT^®^ were viable, and no dead cells were observed. In turn, the cells incubated with DMSO solution (positive control of cytotoxicity) were mainly dead (Figure 7b).

Taking into consideration obtained results (Figure 7a,b), it should be concluded that fibroblast viability is most likely correlated with the ability of biomaterials to release calcium ions to the culture medium (Figure 6). Extract from Cur_11% dressing exhibited the most beneficial effect on fibroblast viability and it contained the highest concentration of calcium ions compared to both the control extract as well as extracts from other biomaterials. Considering the fact that extract obtained from Cur_11% biomaterial enhanced fibroblast viability more potently than other tested biomaterials, it was allocated to the next cell culture experiment—evaluation of cell proliferation.

### 3.8. Fibroblast Proliferation

**Figure 8:**
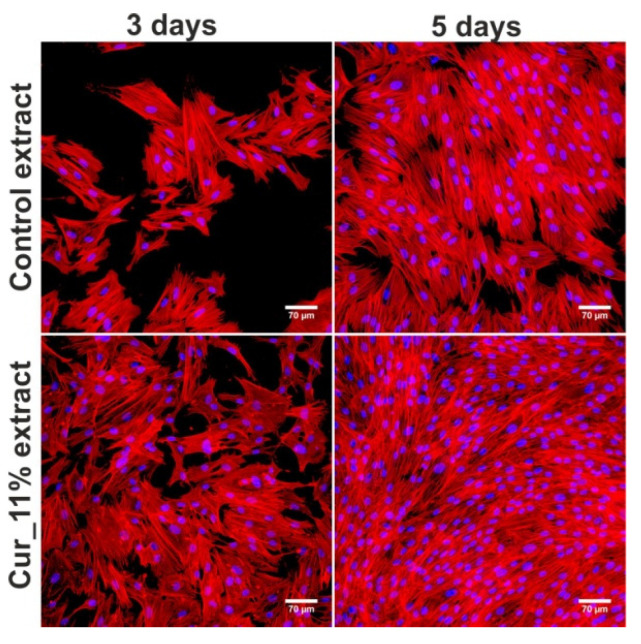
The confocal laser scanning microscope (CLSM) images presenting skin fibroblasts (BJ cell line, ATCC CRL-2522^TM^) after three- and five-day incubation with Cur_11% extract. Culture medium incubated without biomaterial was served as a control. Extracts were prepared in culture medium (EMEM with addition of 10% FBS) according to ISO 10993-5:2009 standard recommendations [37]. Nuclei—blue fluorescence after Hoechst 33342 staining; cytoskeletal filaments—red fluorescence after AlexaFluor^TM^ 635 phalloidin staining; magnification 200×, scale bar equals 70 μm.

The WST-8 assay revealed that extract obtained from Cur_11% biomaterial promoted growth and proliferation of BJ cells compared to the control extract (Table 3). Moreover, the CLSM images (Figure 8) showed that the number of cells treated with Cur_11% extract was higher compared to the number of control cells. Thus, these results are in good agreement with the data obtained during the cell viability evaluation (Figure 7a,b), as such extract also significantly promoted fibroblast viability compared to control extract.

## 4. Conclusions

To sum up, three curdlan-based biomaterials, namely Cur_5%, Cur_8%, and Cur_11%, were fabricated via ion-exchanging dialysis against a CaCl_2_ solution followed by two-step freezing, and subsequent freeze-drying. Combination of these three techniques permitted the production of curdlan-based biomaterials, which exhibited good capacity to absorb simulated wound fluid compared to the commercially available dressing—KALTOSTAT^®^. New curdlan-based biomaterials became beneficial hydrogel structures in the wet state and simultaneously allowed for proper water vapor permeability. Moreover, they possessed the ability to release great amounts of calcium ions to the surrounding environment, which are essential during wound healing. Considering the main requirements for bioactive wound dressings, Cur_11% biomaterial had the most desired properties. Importantly, it possessed similar absorbent properties to KALTOSTAT^®^, but supported fibroblast viability significantly better than this commercial dressing. Cur_11% biomaterial was also found to promote fibroblast proliferation. Thus, Cur_11% hydrogel may be considered as a promising bioactive dressing for wound healing with moderate to high amounts of exudate.

## 5. Patents

The fabrication procedure of curdlan dressings was claimed in the Polish patent application no. 432848 (“The fabrication method of absorbent biomaterial based on curdlan for medical applications”).

## Figures and Tables

**Table 1:** Specific surface area, cumulative pore area, and average pore size of Cur_5%, Cur_8%, and Cur_11% biomaterials.

Parameter	Cur_5%	Cur_8%	Cur_11%
Specific surface area * [m^2^/g] × 10^−2^	100	79.04	50.13
Cumulative pore area ^#^ [m^2^/g] × 10^−2^	69.74	45.60	17.10
Average pore size ^$^ [nm]	14.25	27.30	47.95

* Specific surface area was calculated from N_2_ adsorption isotherms using Brunauer-Emmett-Teller (BET) method; ^#^ Cumulative pore area was determined from Barrett-Joyner-Halenda (BJH) adsorption cumulative surface area of pores between 1.70 nm and 300.00 nm diameter; ^$^ Average pore size was determined from Barrett-Joyner-Halenda (BJH) adsorption average pore width (4 V/A).

**Table 2:** The values of Young’s modulus and tensile strength of Cur_5%, Cur_8%, and Cur_11% after 24 h incubation in simulated wound fluid (SWF).

Biomaterial	Young’s Modulus ± SD [MPa]	Tensile Strength ± SD [MPa]
Cur_5%	0.179 ± 0.036 *	0.029 ± 0.002 *
Cur_8%	0.189 ± 0.004 *	0.033 ± 0.011 *
Cur_11%	0.296 ± 0.036	0.051 ± 0.008

* significantly different results compared to Cur_11% biomaterial (unpaired *t*-test, *p* < 0.05).

**Table 3:** Influence of Cur_11% extract and control extract (culture medium incubated without biomaterial) on skin fibroblast proliferation (BJ cell line, ATCC CRL-2522^TM^). Extracts were prepared in culture medium (EMEM with addition of 10% FBS) according to ISO 10993-5:2009 standard recommendations [37]. The results were expressed as values of fold increase in cell proliferation based on data obtained using WST-8 assay after 3- and 5-day incubation.

Sample	Fold Increase in Cell Proliferation
Control extract	0.79
Cur_11% extract	1.16

## Data Availability

Data available on reasonable request.
